# Supplementary material for: Nearly 20 Years of Genetic Diversity and Evolution of Porcine Circovirus-like Virus P1 from China
Source: Viruses. 2022 Mar 28;14(4):696. doi: 10.3390/v14040696 (PMC9030576; doi:10.3390/v14040696)
Supplement: Supplementary file 1 [file viruses-14-00696-s001.zip › viruses-1607832-supplementary.pdf]

Table S1. Summary of obtained P1 strains in this study.

No.	GenBank accession no.	Strain name	Collection date	Host	length
1	EF514716	HB1	2005	Pig	648
2	JF803741	Czly	2009	Pig	648
3	JF918544	Jiangsu/2010	2010	Pig	648
4	JN040276	NJLH	2011	Pig	648
5	JN040277	HAHZ	2011	Pig	648
6	JN040278	CZJT	2011	Pig	648
7	JN040279	CHAH	2010	Pig	648
8	JN104035	CHHEN	2011	Pig	648
9	JN104036	NTTZ	2010	Pig	648
10	JN104037	WXYX	2010	Pig	648
11	JN104038	CHHEB	2011	Pig	648
12	JN104039	JSHA	2010	Pig	648
13	JN207908	ZJJH	2011	Pig	648
14	JN207909	AHSZ	2011	Pig	648
15	JN207910	JSLYG	2010	Pig	648
16	JN207911	ZJQZ	2011	Pig	648
17	JN207912	ZJSX	2009	Pig	648
18	JN207913	ZJHZ	2010	Pig	648
19	JN207914	ZJNB	2011	Pig	648
20	JN207915	CHHEB2	2011	Pig	648
21	KJ612072	JSNJ	2014	Pig	648
22	KU143774	JSXZ	2015	Pig	648
23	KU243695	JSPZ	2015	Pig	648
24	KU254645	HBA01	2015	Pig	648
25	KU323639	SYD1207	2015	Pig	648
26	KU323640	NSd29	2015	Pig	648
27	KU324510	NSE02	2015	Pig	648
28	KU324511	YX35FX	2015	Pig	648
29	KU350633	YX25	2015	Pig	648
30	KU350634	BYXT	2015	Pig	648
31	KU356936	4PZ-1	2015	Pig	648
32	KU356937	2PZ-1	2015	Pig	648
33	KU356938	E061362	2015	Pig	648
34	KU356939	E051344	2015	Pig	648
35	KU356940	PZ4S20151025FX	2015	Pig	648
36	KU356941	PZ1F20151025	2015	Pig	648
37	KY462783	JSDY	2016	Cattle	648
38	KY462784	JSJN	2016	Cattle	648
39	KY462785	JSNJ	2016	Rabbit	648
40	KY462786	JSZJ	2016	Rabbit	648
41	KY462787	JSXZ	2016	Goat	648

42	KY462788	JSLYG	2017	Goat	648
43	MF716583	PZ1	2016	Pig	648
44	MF716584	PZ2	2016	Pig	648
45	MF716585	PZ3	2016	Pig	648
46	MF802833	SQ	2017	Pig	648
47	MG708301	ZJK01	2017	Pig	648
48	MG708302	ZJK02	2017	Pig	647
49	MG708303	ZJK03	2017	Pig	648
50	MG708304	QHD	2017	Pig	648
51	MG708305	BD	2017	Pig	648
52	MG708306	SJZ	2017	Pig	648
53	MG708307	XT01	2017	Pig	648
54	MG708308	XT02	2017	Pig	648
55	MG708309	XT	2017	Pig	648
56	MG708310	HS01	2017	Pig	648
57	MG708311	HS02	2017	Pig	648
58	MG708312	CZ	2017	Pig	648
59	MH167398	HeB01	2013	Pig	648
60	MH167399	HeB02	2014	Pig	648
61	MH167400	HeN01	2013	Pig	648
62	MH167401	SD01	2014	Pig	648
63	MH167402	AnH01	2013	Pig	648
64	MH167403	AnH02	2014	Pig	648
65	MH167404	ZhJ01	2014	Pig	648
66	MH167405	JS01	2013	Pig	648
67	MH379143	NJ01	2018	Dog	648
68	MH379144	NJ02	2018	Dog	648
69	MH445290	HuN01	2017	Pig	648
70	MH445291	HuN02	2017	Pig	648
71	MH445292	HuN03	2017	Pig	648
72	MH445293	HuN04	2018	Pig	648
73	MT318811	HeB01	2019	Dog	648
74	MT318812	HeB02	2019	Dog	648
75	MT318820	NJ01	2018	Cat	648
76	MT318821	NJ02	2018	Cat	648
77	MT318822	NJ03	2019	Cat	648
78	MT318823	HeB01	2018	Cat	648
79	MT318824	HeB02	2019	Cat	648
80	MW263905	Tibet-P1	2020	Yak	648
81	NC036593	PZ1	2016	Pig	648
82	OL581721	JS02c	2014	Pig	649
83	OL581722	JS03c	2004	Pig	649
84	OL581723	HuN06c	2017	Pig	649
85	OL792747	JS20-1	2020	Pig	648

86	OL792748	JS20-2	2020	Pig	648
87	OL792749	JS20-3	2020	Pig	648
88	OL792750	JS20-4	2020	Pig	648
89	OL792751	JS20-5	2020	Pig	648
90	OL792752	JS20-6	2020	Pig	648
91	OL792753	JS20-7	2020	Pig	648
92	OL792754	JS20-8	2020	Pig	648
93	OL792755	JS20-9	2020	Pig	649
94	OL792756	JS21-1	2021	Pig	648
95	OL792757	JS21-2	2021	Pig	648
96	OL792758	JS21-3	2021	Pig	648
